# Receptor-mediated signaling at plasmodesmata

**DOI:** 10.3389/fpls.2013.00521

**Published:** 2013-12-18

**Authors:** Christine Faulkner

**Affiliations:** John Innes Centre, Norwich Research ParkNorwich, UK

**Keywords:** plasmodesmata, receptor kinase, receptor protein, lipid raft, tetraspanin enriched microdomain

## Abstract

Plasmodesmata (PD) generate continuity between plant cells via the cytoplasm, endoplasmic reticulum (ER) and plasma membrane (PM), allowing movement of different classes of molecules between cells. Proteomic data indicates that the PD PM hosts many receptors and receptor kinases, as well as lipid raft and tetraspanin enriched microdomain associated proteins, suggesting the hypothesis that the PD PM is specialized with respect to both composition and function. PD-located receptor proteins and receptor kinases are responsible for perception of microbe associated molecular patterns at PD and initiate signaling that mediates changes to PD flux. In addition, developmentally relevant receptor kinases have different interactions dependent upon whether located at the PD PM or the cellular PM. The implications of these findings are that receptor-mediated signaling in PD membranes differs from that in the cellular PM and, in light the identification of PD-located proteins associated with membrane microdomains and the role of membrane microdomains in analogous signaling processes in animals, suggests that the PD PM contains specialized signaling platforms.

## INTRODUCTION – PLASMODESMAL STRUCTURE AND FUNCTION

Plant cells are connected to their neighbors via structural channels called plasmodesmata (PD), allowing the movement of molecules between cells and tissues. Molecular flux via PD is essential for many processes requiring intercellular communication and regulation of PD function can control the timing of signaling between cells in these contexts. PD are plasma membrane (PM) lined channels that cross the cell wall generating cytoplasmic and PM continuity between cells (Maule et al., 2012). The endoplasmic reticulum (ER) also passes from cell to cell via PD and the PD ER is known as the desmotubule. Trafficking from cell-to-cell has been observed to occur via the cytoplasmic channel (Oparka and Prior, 1988; Tucker et al., 1989), the desmotubule lumen (Barton et al., 2011), and the desmotubule membrane (Grabski et al., 1993; Martens et al., 2006; Guenoune-Gelbart et al., 2008), offering avenues for both soluble and lipid-based transport.

Our current understanding of PD function and the regulation of PD flux is limited. It has been established that PD allow the passage of molecules that are small enough to diffuse through the cytoplasmic sleeve and the current hypothesis is that dynamic regulation of the sleeve size dictates the size exclusion limit (SEL) for such molecules. This non-specific transport between cells is thought to be primarily regulated by the abundance of callose in the cell wall surrounding the necks of the channel (Zavaliev et al., 2011). Callose deposition pushes the PM inward to reduce the sleeve size and thus localized callose synthesis and hydrolysis regulates the flux of molecules through the channel (Maule et al., 2012). Specific and/or active transport between cells is possible for larger molecules such as transcription factors and viruses. In the case of the KNOX family transcription factors, this transport is mediated by a chaperonin which is required to unfold the transcription factor after passage through the PD channel, implicating protein folding as an essential component of the translocation mechanism (Xu et al., 2011). However, both KNOTTED1 (Lucas et al., 1995) and viral movement proteins (Wolf et al., 1989) can increase the PD SEL so it remains possible that this active and specific trafficking process also involves some alteration to PD structure.

Proteins located at PD are likely to have functions specific to the regulation and structure of PD. Recent work has identified several receptor kinases and receptor proteins that are specifically located, or function, at the PD PM (Thomas et al., 2008; Fernandez-Calvino et al., 2011; Lee et al., 2011; Faulkner et al., 2013; Stahl et al., 2013), in addition to callose synthases (Guseman et al., 2010; Vaten et al., 2011) and β-1,3-glucanases (Levy et al., 2007; Benitez-Alfonso et al., 2013) that mediate callose turnover specifically at PD. While the intermediate signaling steps are unknown, these results indicate that receptors exposed at the PD membrane perceive changes to the cellular environment and initiate a downstream cascade that ultimately regulates PD SEL and intercellular transport.

## MEMBRANE MICRODOMAINS ARE PLATFORMS FOR RECEPTOR-MEDIATED SIGNALING IN ANIMAL CELLS

Subdivision of the PM into microdomains is required for membrane located processes in a variety of systems. In mammalian cells, membrane compartmentalization and microdomains define signaling processes that include B-cell and T-cell activation, apoptosis and insulin signaling. In these contexts, both lipid rafts and tetraspanin enriched microdomains (TEMs) alter signaling activity of specific receptors located in the PM. Lipid rafts and TEMs are microdomains of the PM that are differentiated by their lipid and protein composition. Lipid rafts are enriched in cholesterol and glycoshingolipids, and proteins such as stomatin/prohibitin/flotillin/HflK/C (SPFH) domain proteins. In plants, lipid rafts are also defined by the presence of the plant-specific protein remorin (Jarsch and Ott, 2011). Like lipid rafts, TEMs are discrete areas of membrane but are defined by an enrichment of tetraspanin proteins. The difference in lipid and protein composition of membrane microdomains means they exhibit varying resistance to detergents. This allows the extraction of many raft and TEM components in detergent resistant membrane (DRM) fractions, although the biological accuracy of this fraction as corresponds to raft identity is a matter of some debate.

Tetraspanin enriched microdomains and lipid rafts act as platforms for receptor-mediated signaling in a number of contexts. In mammals, B-cell activation relies on the detection of an antigen by B-cell receptor (BCR) microclusters and BCR signaling occurs via the co-receptor CD19. CD19 is organized and immobilized in the membrane by the tetraspanin CD81 (Mattila et al., 2013). *Cd81*^-/-^ mutant cells are deficient in downstream events such as effector phosphorylation (Mattila et al., 2013) illustrating that the membrane compartmentalization of CD19 and BCR is fundamental to the process of B-cell activation. TEMs also play a role in pattern recognition receptor (PRR) display and signaling. For example, the tetraspanin CD37 interacts with the β-glucan receptor Dectin-1 in antigen presenting cells and mediates induction of the defense-associated molecule interleukin-6 (Meyer-Wentrup et al., 2007).

In a similar fashion, lipid rafts provide an alternate membrane environment for receptor signaling. One such example is the regulation of apoptosis in mammalian cells by lipid raft localized signaling. Ligand-independent activation of apoptotic signaling by the tumor necrosis factor protein Fas involves oligomerization of the receptor in lipid rafts and subsequent recruitment of other components of the death-inducing signaling complex that triggers Caspase-8 activity and apoptotic signaling (George and Wu, 2012).

The common theme to the involvement of lipid rafts and TEMs in signaling is the spatial concentration (or separation) of signaling components. It seems likely that while there is little understanding of the primary functions of membrane microdomains in plant cells, lipid rafts and TEMs might facilitate signaling in a similar manner.

## MEMBRANE MICRODOMAINS AT PD

Recent studies have identified that the protein composition of the PD PM differs from the cellular PM, with the PD PM containing a number of unique or enriched proteins (Thomas et al., 2008; Fernandez-Calvino et al., 2011; Stahl et al., 2013). Correspondingly, it is likely that the lipid composition of the PD PM also differs from the cellular PM and the possibility that the PD PM contains lipid rafts was raised by the localization of remorin to the PD PM (Raffaele et al., 2009). Remorin has a functional role in PD trafficking as the *Solanum tuberosum* Remorin (REM) 1.3 regulates trafficking of potato virus X (PVX) from cell-to-cell in tobacco (Raffaele et al., 2009). In *Arabidopsis*, *At*REM1.2 was identified in the PD proteome along with a number of SPFH domain proteins (Fernandez-Calvino et al., 2011), further suggesting the existence of lipid rafts in the PD PM.

Stomatin/prohibitin/flotillin/HflK/C domain proteins are found in lipid rafts in membranes in mammalian systems where they are associated with the compartmentalization of membranes, ion channel regulation, membrane trafficking and connection of membranes to the cytoskeleton (Browman et al., 2007). In plants, several SPFH proteins have been characterized and, as in mammalian systems, these proteins appear to have roles in the definition and activity of membrane domains. The *Arabidopsis* SPFH domain protein FLOTILLIN1 (FLOT1) was recently shown to function in clathrin-independent endocytosis, and immunogold labeling of the PM indicated that FLOT1 clustered in the PM in a manner consistent with its localization in microdomains (Li et al., 2012). Similarly, FLOT2 and FLOT4 are unevenly distributed in *Medicago* root cells (Haney and Long, 2010). The *Arabidopsis* HYPERSENSITIVE INDUCED REACTION PROTEINS (*At*HIR) are SPFH domain proteins and both *At*HIR1 and *At*HIR2 interact with the resistance protein RPS2. This interaction is required for defense responses triggered by RPS2 and occurs unevenly in the PM, suggestive of localization of activity in membrane sub-domains (Qi et al., 2011). The PD proteome contains the SPFH domain proteins *At*HIR1-4, FLOT1, a stomatin-like protein, an erlin-2-like protein and PROHIBITIN3 and 7 (Fernandez-Calvino et al., 2011). The association of SPFH domain proteins with lipid rafts, and their putative association with the PD PM, further allows the hypothesis that lipid rafts in the PD PM create PD-specific signaling platforms.

The identification of TETRASPANIN3 (TET3) in the PD proteome suggests that the PD PM also houses TEMs in addition to lipid rafts. TET3 was confirmed as a PD-located protein by subcellular localization of a TET3-YFP fusion (Fernandez-Calvino et al., 2011). There is scarce information relating to the abundance and function of TEMs in plant cells but recent characterization of the subcellular localization of a number of *Arabidopsis* tetraspanins provided some evidence that tetraspanins do associate with membrane microdomains in plant cells like in mammalian cells (Boavida et al., 2013). The localization pattern of TET5 was consistent with it being a PD-associated protein, but as yet no functional role in PD-specific membrane microdomains has been determined for either TET5 or TET3.

## PROTEIN MICRODOMAINS AT PD

PLASMODESMATA LOCATED PROTEINS (PDLPs) were identified as an 8-member family of novel receptor proteins that are located at the PD PM (Thomas et al., 2008). PDLPs have two extracellular DUF26 domains, a transmembrane domain and a short cytoplasmic tail with the transmembrane domain of PDLP1 sufficient to convey PD targeting of a fluorescent reporter (Thomas et al., 2008). This suggests that PDLP1 is anchored at PD via its interaction with the membrane environment, either with another PD PM protein or with the membrane phospholipids present at the PD PM.

The specificity of PDLP localization indicates that the PD PM is differentiated from the cellular PM but in addition to this there is evidence that the PD PM is further subdivided into microdomains. While PDLPs were immunolocalized to the central PD PM, another PD PM associated protein, PLASMODESMATA CALLOSE BINDING1 (PDCB1, was immunolocalized to the PD PM at the neck of the channel (Maule et al., 2011). Given the callose binding capacity of PDCB1 it is consistent that this protein is located at a site of callose deposition, but it should also be noted that PDCB1 is a glycophosphatidylinositol (GPI) anchored protein. GPI anchored proteins are tethered to the PM, preferentially at lipid rafts (Mayor and Riezman, 2004). Thus, considering the preference for localization of GPI anchored proteins within lipid rafts, it is possible that PD PM subdomains correspond with lipid rafts and/or TEMs.

## RECEPTOR MEDIATED SIGNALING AT THE PD PM

Protein localization to and within the PD PM must hold functional significance for the mode of activity of proteins which show PD specificity. Accordingly, PD PM protein and membrane microdomains are likely to be fundamental to PD function. The observation that the lipid raft protein *St*REM1.3 has the capacity to directly bind the PVX TRIPLE GENE BLOCK1 protein and to regulate the trafficking of the virus from cell to cell (Raffaele et al., 2009) supports this hypothesis. It seems likely that PD PM microdomains contribute to the regulation of PD in multiple ways.

As described above, lipid rafts and TEMs in mammalian cells often function in receptor display and activation, providing a platform for specialized and localized signaling. This has particular relevance to receptor signaling in mobile immune cells, as illustrated by membrane microdomain involvement in B cell activation. At first glance, immune responses in plant cells have fundamental differences to those in animal cells as in plants each cell must be capable of initiating a response rather than being mediated by an army of specialized, mobile cells. However, in plant cells, early pathogen perception and defense responses are mediated by receptor kinases and receptor proteins exposed at the cell surface as in animal cells. These receptors trigger a medley of intracellular signaling events that launch defense responses.

Like for mammalian cells, lipid rafts have been associated with defense signaling in plant cells. For example, following treatment of *Arabidopsis* cell suspension cultures with the bacterial flagellin derivative flg22, a number or receptor kinases and other signaling proteins were enriched in DRM fractions (Keinath et al., 2010). These included the flagellin perceiving receptor kinase FLAGELLIN SENSING2 (FLS2) suggesting compartmentalization of this PRR in the PM. FLS2 also co-immunoprecipitates with the SPFH domain protein *At*HIR2 (Qi and Katagiri, 2012), providing further evidence that FLS2 activity associates with lipid rafts. This allows speculation that FLS2 activity, and that of other plant receptor kinases, is facilitated by recruitment to membrane microdomains like is seen in animal cells.

**FIGURE 1:**
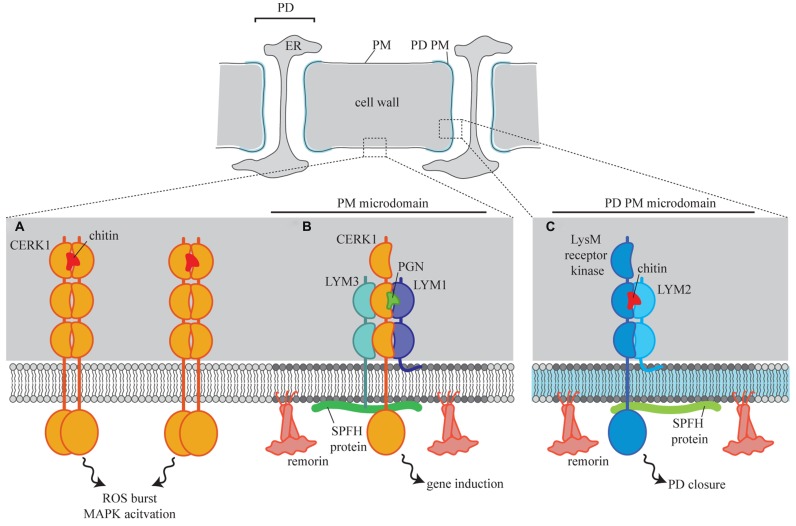
**A model for the spatial separation of LysM receptor signaling in plant cells.** In the cellular PM CERK1 perceives chitin and triggers a number of defense responses intracellularly **(A)**. Chitin perception at the PD PM **(C)** requires the GPI-anchored receptor protein LYM2, via interaction with an unknown LysM receptor kinase. Remorin and SPFH proteins in the PD PM define lipid rafts which act as platforms for this signaling complex. In the cellular PM CERK1 is recruited to lipid rafts where it interacts with LYM1 and LYM3 to form a peptidoglycan (PGN) perception complex **(B)**.

Recent work has identified PD PM located proteins that play a role in the regulation of intercellular flux during defense responses. *Arabidopsis* LYSIN MOTIF DOMAIN-CONTAINING GLYCOSYLPHOSPHATIDYLINOSITOL-ANCHORED PROTEIN 2 (*At*LYM2) is a PD-located, GPI-anchored receptor protein that perceives chitin and in response triggers PD closure (Faulkner et al., 2013). Significantly, *At*LYM2-mediated chitin perception and signaling occurs independently of other chitin-triggered responses such as an increase in reactive oxygen species (ROS burst) or mitogen activated protein kinase (MAPK) activation. Chitin-triggered ROS burst and MAPK activation are mediated by the receptor kinase CHITIN ELICITOR RECEPTOR KINASE1 (CERK1; Miya et al., 2007) which is present in the cellular PM and dimerizes in the presence of chitin (Liu et al., 2012). CERK1 is not required for chitin-triggered PD closure and therefore there is functional and spatial separation of chitin-triggered defense. Given that *At*LYM2 has a GPI anchor, and that the PD PM likely contains membrane microdomains, it is tempting to speculate that this difference in signaling location is facilitated by differential association of the relevant receptors with PD PM lipid rafts (**Figure 1**). CERK1 also forms a complex with the receptor proteins *At*LYM1 and *At*LYM3 for the perception of peptidoglycan (Willmann et al., 2011). Again, considering that *At*LYM1 is a GPI anchored protein, this complex formation might be mediated by recruitment of CERK1 to a different PM microdomain in the presence of peptidoglycan (**Figure 1**).

Recently, we showed that in addition to mediating flagellin triggered defense responses such as ROS burst and MAPK activation, FLS2 mediates flg22 induced closure of PD (Faulkner et al., 2013). A FLS2-GFP fusion is located at the PD PM as well as the cellular PM, suggesting that, like LYM2, it could trigger a site specific response. Upon binding of flg22, FLS2 forms a complex with another receptor kinase BRI1 ASSOCIATED RECEPTOR KINASE1 (BAK1) and this interaction is required for the initiation of FLS2 signaling cascades (Monaghan and Zipfel, 2012). It is not yet known whether PD closure is dependent on FLS2-BAK1 interaction but it is possible that either FLS2, or the FLS2/BAK1 complex, interacts with PD PM specific components that mediate FLS2-triggered PD closure. Given the association of FLS2 with *At*HIR2, and the identification of *At*HIR1-4 as putative PD PM proteins, it is possible that PD localization and signaling of FLS2 also occurs via interaction with lipid rafts at PD.

Plasmodesmata responses in the context of defense have also implicated the activity of PDLP5. *PDLP5* is upregulated in response to salicylic acid (SA) and PDLP5 regulates callose deposition to close PD in response to SA (Lee et al., 2011; Wang et al., 2013). Given that SA biosynthesis is an intracellular process, and that SA regulates *PDLP5* transcription, the role of PDLP5 as a receptor protein in this response is still unclear. Wang et al. (2013) proposed there might be a direct link between PDLP5 and callose synthases. Whether this link comes from direct complex formation between these proteins, or whether PDLP5 activity triggers a signal cascade that results in increased callose synthase activity remains to be determined.

Receptor mediated signaling at the PD PM is unlikely to be unique to defense responses. Two independent proteomic studies identified a number of receptor kinases that reside in the PD PM (Fernandez-Calvino et al., 2011; Jo et al., 2011) and thus it is probable that this membrane domain provides a platform for PD-relevant signaling initiated by a variety of triggers. Compelling evidence to support this comes from the observation that differential receptor-kinase complex formation occurs at the PD PM during the definition of root stemness (Stahl et al., 2013). The receptor kinases CLAVATA1 (CLV1) and ARABIDOPSIS CRINKLY4 (ACR4) are involved in maintenance of the root meristem and can form both homo- and heteromeric protein complexes. While both receptors are present in the PM, ACR4 accumulates at the PD PM relative to the cellular PM. FRET-FLIM experiments allowed the authors to propose that the cellular PM contains CLV1-ACR4 heterodimers and ACR4-ACR4 homodimers while at the PD higher order homo- and heteromeric complexes form due to the higher concentration of ACR4 (Stahl et al., 2013). Cell fate specificity and developmental processes have been shown to depend on the intercellular movement of proteins such as transcription factors in several tissues. The specific PD-associated function of ACR4 and CLV1 has not yet been determined but presumably the higher order complexes of ACR4 and CLV1 in the PD PM mediate PD specific signaling that regulates the PD aperture and the movement of a non-cell autonomous signal that defines root stemness. It is again possible that the concentration of ACR4 in the PD PM, and the formation of a differential signaling platform, is a consequence of specific recruitment of ACR4 to a PD PM microdomain defined by the lipid environment.

## CONCLUSION

There is a significant body of evidence that suggests both lipid rafts and TEMs provide signaling platforms in plant cells. Recent advances have highlighted the specificity of a number of PD PM located receptor proteins and receptor kinases that have PD specific functions. When combined with the identification of a number of lipid raft and TEM associated proteins in the PD PM we can begin to build a model in which PD specific receptors are localized and activated via recruitment to PD PM microdomains. Future work to characterize the composition of the PD PM and the signaling cascades triggered by the resident proteins will elucidate mechanisms of PD function and regulation, allowing a more in depth understanding of intercellular communication and co-ordination.

## Conflict of Interest Statement

The author declares that the research was conducted in the absence of any commercial or financial relationships that could be construed as a potential conflict of interest.
